# The New Scriptures

**Published:** 1875-02

**Authors:** 


### THE NEW SCRIPTURES.

*According to Tyndall, Huxley, Spencer, and Darwin.*

(GENESIS, CHAPTER II.)

1. Primarily the Unknowable moved upon cosmos and evolved protoplasm.

2. And protoplasm was inorganic and undifferentiated, containing all things in potential energy; and a spirit of evolution moved upon the fluid mass.

3. And the Unknowable said, Let atoms attract; and their contact begat light, heat, and electricity.

4. And the Unconditioned differentiated the atoms, each after its kind; and their combinations begat rock, air, and water.

5. And there went out a spirit of evolution from the Unconditioned, and, working in protoplasm by accretion and absorption, produced the organic cell.

6. And cell, by nutrition, evolved primodial germ, and germ developed protogene, and protogene begat eozoön begat monad, and monad begat animalcule.

7. And animalcule begat ephemera; then began creeping things to multiply on the face of the earth.

8. And earthy atom in vegetable protoplasm begat the molecule, and thence came all grass aad every herb in the earth.

9. And animalcula in the water evolved fins, tails, claws, and scales; and in the air, wings and beaks; and on the land they sprouted such organs as were necessary as played upon by the environment.

10. And by accretion and absorption came the radiata and mollusca, and molusca begat articulata, and articulata begat vertebrata.

11. Now these are the generations of the higher vertebrata, in the cosmic period that the Unknowable evoluted the bipedal mammalia,

12. And every man of the earth, while he was yet a monkey, and the horse, while he was a hipparion, before he was an oredon.

13. Out of the ascidian came the amphibian and begat the pentadactyle, and the pentadactyle by inheritance and selection produced the hylobate, from which are the simiadæ in all their tribes.

14. And out of the simiadæ the lemur prevailed above his fellows and produced the platyrrhine monkey.

15. And out of the platyrrhine begat the catarrhine, and the catarrhine monkey begat the anthropoid ape, and the ape begat the longimanous ourang, and the ourang begat the chimpanzee, and the chimpanzee evoluted the what-is-it.

16. And the what-is-it went into the land of Nod and took him a wife of the longimanous gibbons.

17. And in process of the cosmic period were born unto them and their children the anthropomorphic primordial types.

18. The homunculus, the prognathus, the trogolodyte, the autochthon, the terragen—these are the generations of primeval man.

19. And primeval man was naked and not ashamed, but lived in quadrumanous innocence, and struggled mightily to harmonize with the environment.

20. And by inheritance and natural selection did he progress from the stable and homogeneous to the complex and heterogeneous; for the weakest died, and the strongest grew and multiplied.

21. And man grew a thumb, for that he had need of it, and developed capacities for prey.

22. For, behold, the swiftest men caught the most animals, and the swiftest animals got away from the most men; wherefore the slow animals were eaten, and the slow men starved to death.

23. And as types were differentiated, the weaker types continually disappeared.

24. And the earth was filled with violence; for man strove with man, and tribe with tribe, whereby they killed off the weak and foolish, and secured the survival of the fittest.

We much believe in "blood," and think, notwithstanding Messrs. Darwin & Co., that it "will tell" down to the latest generation.

If man was an ape at his creation, is he not an ape still? If, in the processes of "evolution" to which it is said he has been subjected during all the past ages, though he may have assumed various outward forms till he reached his present stature, was the thin fluid sluggishly flowing through the veins of his "lemur" protoplast—the "rich, blue blood" that now leaps in vivid pulsations from the heart of the intellectual Titan, or gentleman to the manner born?

And, as "man strove with man and tribe with tribe, whereby the strong killed off the weak and foolish, and secured the survival of the fittest," did they "kill off" all the weak and foolish, and secure the survival of *only* the fittest? "As the types were differentiated," *did* the weaker types continually disappear, and were the slow animals all eaten, and all the "slow men starved to death?" We guess not, and again beg leave to differ from Mr. Darwin and his coadjutors. We think the "primeval man" is still progressing from "the stable and homogeneous to the complex and heterogeneous;" and though the strongest still grow and multiply, do not the weakest also?

Some men, it seems, cannot be improved. They will mark out a path for themselves to walk in, from which they will turn neither to the right nor left, and will insist upon others treading the same beaten track, or endure their anathemas.

A grand discovery in some science, that will give a glorious impetus to the onward march of the age, is to them all bosh, or only "a new-fangled notion;" if a contemporary advances a theory, and one reduced to practical utility, because it is *new*, and is not theirs, the bold champion of progress is smitten by their oracular *fulmen brutum;* and because they cannot or will not understand, and did not inaugurate the ethics of a medical organization, or the jurisprudence of some church government, the adherents of such fraternities are prescribed, and cast forth from the synagogue.

The gentlemen of one dogma of their own are not as accommodating as the young man who applied to the district school committee to teach the "young idea" of the neighborhood; and upon being asked by the said committee what was the form of the earth, replied that some said it was flat, and others said it was round, but as for his part, he was willing to teach either way. With these men of the "weaker type," in their dogmatic and lucid ratiocination, it is *meum*, and not *teum;* it is "I am right, and you are wrong;" and the difference at last is, as we Georgians would say, only between "we'uns and you'uns."

As to the preceding and capital "scientific *jeux d'esprit*," which so happily takes off the much talked-of science of "evolution," etc., it is a notable fact that, as Virchow says, the materialistic doctrine "denies its own dogmatism, and appears in the garb of science—that it professes to rest on fact, when it is but speculation, and attempts to annex territories to the domain of natural science before they have been fully conquered." All the sophistry of the most subtle scientific theories can never destroy that strong and sure foundation, "In the beginning God created the heavens and the earth," nor overshadow the Divine mandate and assertion, "Let there be light and there was light." And man, made in God's own image, exulting in the divinity of his origin, would prefer the simple, child-like faith of the Bible to all the vain materialistic speculations of a host of Huxleys, Spencers and their *confreres.* P.

